# Treating severe paediatric asthma with mepolizumab or omalizumab: a protocol for the TREAT randomised non-inferiority trial

**DOI:** 10.1136/bmjopen-2024-090749

**Published:** 2024-08-21

**Authors:** Victoria Cornelius, Daphne Babalis, William D Carroll, Steven Cunningham, Louise Fleming, Erol Gaillard, Atul Gupta, Leila Janani, Erika Kennington, Clare Murray, Prasad Nagakumar, Graham Roberts, Paul Seddon, Ian Sinha, Claire Streatfield, Elise Weir, Sejal Saglani

**Affiliations:** 1 Health and Social Care Research, Imperial College London, London, UK; 2 Imperial Clinical Trials Unit, Imperial College London, London, UK; 3 Imperial College London School of Public Health, London, UK; 4 Academic Department of Child Health, University Hospitals of North Midlands NHS Trust, Stoke-on-Trent, UK; 5 Institute of Applied Clinical Sciences, Keele University, Keele, UK; 6 Department of Child Life and Health, Royal Hospital for Sick Children, Edinburgh, UK; 7 Royal Brompton Hospital and National Heart & Lung Institute, Imperial College London, London, UK; 8 Leicester NIHR Biomedical Research Centre (Respiratory Theme), Leicester, Leicestershire, UK; 9 Paediatric Clinical Investigation Centre, Leicester, UK; 10 King’s College Hospital Foundation Trust, London, UK; 11 Faculty of Medicine, Imperial College London, London, UK; 12 Asthma and Lung UK, London, UK; 13 Division of Immunology, Immunity to Infection and Respiratory Medicine (DIIIRM), School of Biological Sciences, Faculty of Biology, Medicine and Health, University of Manchester, Manchester, UK; 14 Institute of Inflammation and Aging, Birmingham Women’s and Children’s Hospitals NHS Foundation Trust, Birmingham, UK; 15 University of Birmingham, Birmingham, UK; 16 Human Development and Health Academic Unit, Faculty of Medicine, University of Southampton, Southampton, UK; 17 Respiratory Biomedical Research Unit, Southampton University Hospitals Trust, Southampton, UK; 18 Brighton and Sussex Medical School, Brighton, UK; 19 Faculty of Health and Life Sciences, University of Liverpool, Liverpool, UK; 20 Royal Hospital for Children, Glasgow, UK; 21 National Heart and Lung Institute, Faculty of Medicine, Imperial College London, London, UK

**Keywords:** Paediatric thoracic medicine, Randomized Controlled Trial, Respiratory Function Test, Clinical trials, Asthma

## Abstract

**Introduction:**

A minority of school-aged children with asthma have persistent poor control and experience frequent asthma attacks despite maximal prescribed maintenance therapy. These children have higher morbidity and risk of death. The first add-on biologic therapy, omalizumab, a monoclonal antibody that blocks immunoglobulin (Ig)E, was licensed for children with severe asthma in 2005. While omalizumab is an effective treatment, non-response is common. A second biologic, mepolizumab which blocks interleukin 5 and targets eosinophilic inflammation, was licensed in 2018, but the licence was granted by extrapolation of adult clinical trial data to children. This non-inferiority (NI) trial will determine whether mepolizumab is as efficacious as omalizumab in reducing asthma attacks in children with severe therapy resistant asthma (STRA) and refractory difficult asthma (DA).

**Methods and analysis:**

This is an ongoing multicentre 1:1 randomised NI open-label trial of mepolizumab and omalizumab. Up to 150 children and young people (CYP) aged 6–17 years with severe asthma will be recruited from specialist paediatric severe asthma centres in the UK. Prior to randomisation, children will be monitored for medication adherence for up to 16 weeks to determine STRA and refractory DA diagnoses. Current prescribing recommendations of serum IgE and blood eosinophils will not influence eligibility or enrolment. The primary outcome is the 52-week asthma attack rate. Bayesian analysis using clinician-elicited prior distributions will be used to calculate the posterior probability that mepolizumab is not inferior to omalizumab. Secondary outcomes include Composite Asthma Severity Index, Paediatric Asthma Quality of Life Questionnaire, lung function measures (forced expiratory volume in one second (FEV1), bronchodilator reversibility), fractional exhaled nitric oxide, Asthma Control Test (ACT), health outcomes EuroQol 5 Dimension (EQ-5D) and optimal serum IgE and blood eosinophil levels that may predict a response to therapy. These outcomes will be analysed in a frequentist framework using longitudinal models.

**Ethics and dissemination:**

The study has been approved by the South Central—Berkshire Research Ethics Committee REC Number 19/SC/0634 and had Clinical Trials Authorisation from the Medicines and Healthcare Products Regulatory Agency (MHRA) (EudraCT 2019-004085-17). All parents/legal guardians will give informed consent for their child to participate in the trial, and CYP will give assent to participate. The results will be published in peer-reviewed journals, presented at international conferences and disseminated via our patient and public involvement partners.

**Trial registration number:**

ISRCTN12109108; EudraCT Number: 2019-004085-17.

**STRENGTHS AND LIMITATIONS OF THIS STUDY**

- Randomised head-to-head study of mepolizumab versus omalizumab in children and young people (CYP) with severe therapy resistant asthma (STRA) and refractory difficult asthma (DA) with embedded mechanistic study to provide valuable insight into the mechanism of each treatment.
- Use of a Bayesian analysis with clinician elicited priors for phase II trial and inference framework using posterior probabilities in an uncommon condition.
- Trial to include a rigorous run-in period to accurately diagnose STRA and refractory DA.
- Assessment of the effect of omalizumab and mepolizumab regardless of serum immunoglobulin E and blood eosinophils, to determine optimal biomarkers for CYP.
- Due to differing dose scheduling for the two interventions, the trial is open-label.

## Introduction

 Over 1 million children in the UK are diagnosed with asthma.^1^ Although over 95% of school-aged children with asthma can be controlled with relatively low and safe doses of maintenance inhaled corticosteroids, there is a minority who have persistent poor control and/or frequent asthma attacks despite maximal prescribed maintenance therapy. This group with problematic severe asthma (PSA) presents a significant clinical challenge, as they experience marked morbidity,^2^ use more than 50% of all healthcare resources for asthma^3 4^ and are at increased risk of asthma death.^5^ Improving control and reducing risk for children with PSA is therefore an urgent unmet clinical need.^6^

Omalizumab is one of only three licenced add-on therapies (‘biologic’) for children aged 6–16 years with severe asthma. Its mechanism of action is neutralisation of circulating free immunoglobulin (Ig)E, which leads to reduction in the quantity of cell-bound IgE, downregulation of high-affinity IgE receptors and, eventually, prevention of mediator release from effector cells.^7^ Increasingly, phenotype-directed therapies, specifically therapies that target eosinophilic inflammation, are emerging for use in adult severe asthma.^8^ One of these, mepolizumab, was licenced for use in children aged 6 years and over in Europe in August 2018, but neither its efficacy nor mechanism of action in paediatric severe asthma populations was known when licence was approved. Given the differences between paediatric and adult severe therapy resistant asthma (STRA), it may not be appropriate to extrapolate findings from adult studies into children. There has been one recent trial of mepolizumab efficacy in children aged 6–11 years.^9^ However, this included children with moderate to severe asthma, and focused on children of black and Hispanic ethnicity. There was a reduction in annual number of asthma exacerbations from 1.3 in the placebo group to 0.96 in the mepolizumab group; however, this 27% reduction was only half that seen in previous adult studies.^10^

We will undertake a parallel arm randomised Bayesian non-inferiority (NI) trial to determine how mepolizumab compares to omalizumab in reducing asthma attacks in children with STRA and Refractory difficult asthma (DA).

## Methods and analysis

### Objectives

Our primary objective is to determine whether mepolizumab is as efficacious as omalizumab in reducing asthma attacks over 52 weeks in children and young people (CYP) aged 6–16 years with STRA and refractory DA.

We will also examine the biomarkers by which the effects of mepolizumab and omalizumab are mediated. Specifically, we will measure change in blood eosinophils, serum and sputum eosinophil peroxidase levels for mepolizumab, and change in serum IgE for omalizumab. We will relate reduction in asthma exacerbations to blood eosinophils prior to treatment with mepolizumab and serum IgE prior to treatment with omalizumab.

### Trial design

**Figure 1:**
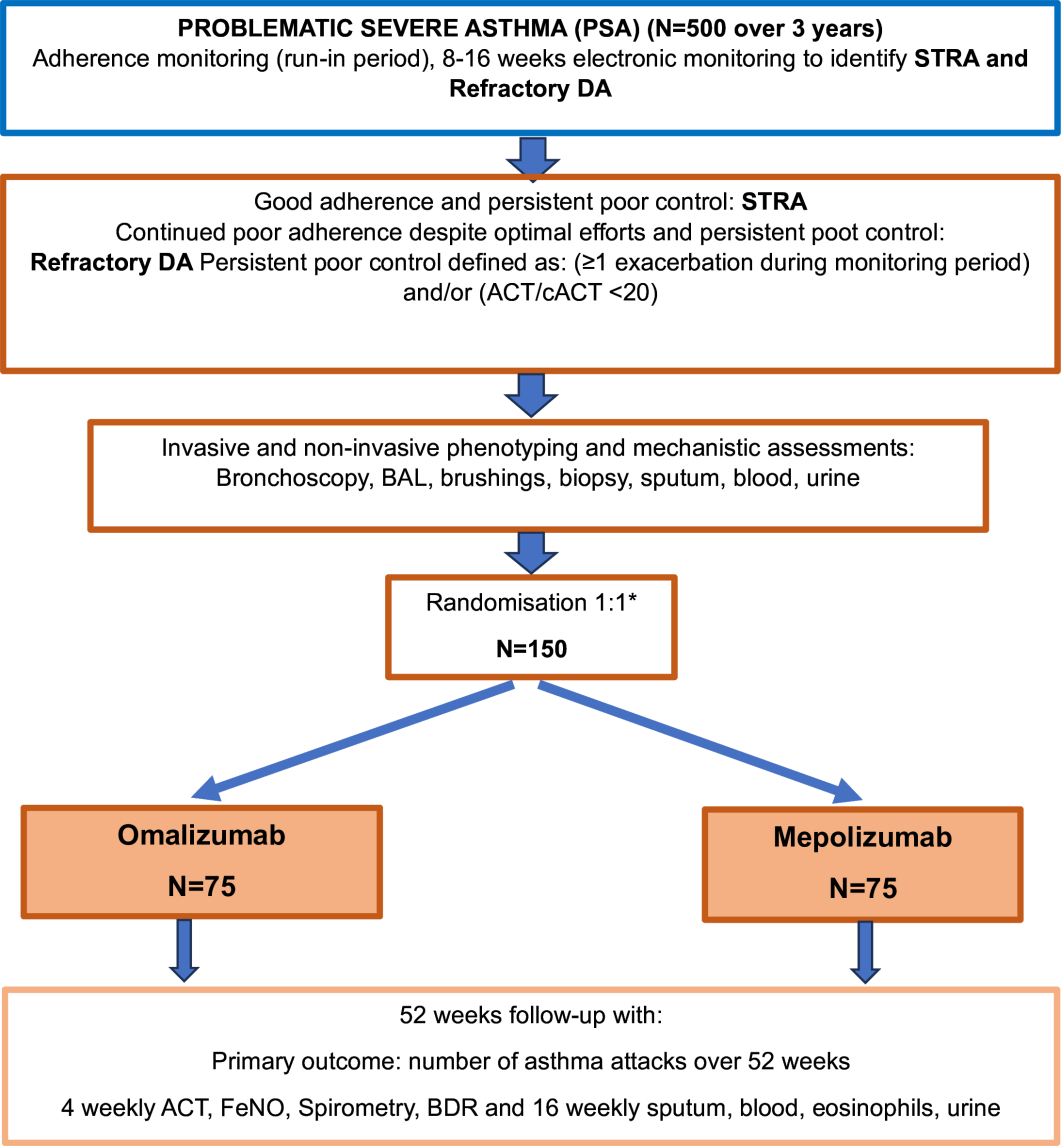
TREAT trial design and study flowchart. *Randomisation (minimisation) stratified by blood eosinophils (</> 300 per µL). IgE (<30, 30–1500, >1500 IU/mL), STRA/refractory DA. ACT, Asthma Control Test; cACT, Childhood Asthma Control Test; DA, difficult asthma; FeNO, fractional exhaled nitric oxide; BAL, bronchoalveolar lavage; BDR, bronchodilator reversibility.

A 52-week multicentre, parallel-arm, 1:1 randomised, open-label, Bayesian NI trial of mepolizumab against omalizumab in up to 150 CYP, with a rigorously monitored run-in period of up to 500 CYP to determine adherence to current asthma treatment, and thereby differentiate true STRA and refractory DA. The study flow is shown in figure 1.

### Eligibility criteria and recruitment

#### Eligibility for run-in study

CYP with PSA as defined below, aged 6–16 years, will be recruited from UK specialist paediatric severe asthma centres to a run-in study to identify if they have STRA or refractory DA. They will be eligible for the run-in phase if they have a confirmed diagnosis of asthma* and poor asthma control** despite being prescribed high dose therapy***.^11^

*Where asthma diagnosis is defined as: documented wheeze plus one or more of:

- Airway hyper-responsiveness confirmed by direct or indirect challenge.
- Documented bronchodilator reversibility (≥12%).
- Recorded evidence of spontaneous variation in forced expiratory volume in one second (FEV1) (≥12%) or peak flow (≥20%) in the past year.

**And poor control is defined as at least 1 of the following:

- Recurrent severe asthma attacks in the past year (≥4 per year if on high dose inhaled corticosteroids OR>2 per year if on maintenance oral corticosteroids) requiring either asthma-related hospital admission (≥4 hours in hospital) or high dose systemic steroids.
- A single PICU admission in the past year.

***And high dose therapy is defined as either of:

- Maintenance inhaled corticosteroids (budesonide≥800 µg/day or fluticasone≥500 µg/day) or equivalent (as defined in the British Thoracic Society/Scottish Intercollegiate Network (BTS/SIGN) guidelines 2019) plus a long-acting β2 agonist plus montelukast (or previous failed trial) or trial of other add-on therapy such as theophylline.
- Maintenance daily or alternate day oral corticosteroids.

During the run-in period, CYP will undertake a period of adherence monitoring using electronic monitoring devices. Those with ongoing poor control (Asthma Control Test (ACT)/Childhood ACT (cACT)<20) and monitored adherence of <80% will enter a period of enhanced monitoring or be withdrawn. CYP with ongoing poor control will be eligible for the randomised controlled trial (RCT) (see online supplemental figure 1 for overview of run-in study).

#### Eligibility for RCT

We will recruit CYP aged 6–17 years with confirmed diagnosis of asthma with:

1. Persistent poor control and/or ≥1 attack after adherence assessment with ≥80% adherence during run-in (STRA).
2. Persistent poor control^+^ and poor adherence despite optimal efforts to improve adherence, including enhanced monitoring (refractory DA).

(see figure 1 for overview of study flow and trial design and full exclusion/inclusion criteria in online supplemental section 1).

^+^Persistent poor control defined as at least one of the following:

- ACT or cACT Score of <20.
- ≥1 severe attack requiring either asthma-related hospital admission (≥4 hours in hospital) or high dose systemic corticosteroids during the adherence monitoring period.

### Setting, recruitment and consent

CYP will be recruited from at least 11 specialist paediatric severe asthma centres in the UK. Identification of eligible CYP may also occur at patient identification centres located close to the specialist centres. CYP may also be recruited via a range of recruitment methods including via the TREAT trial website, advertisements/posters in general practitioner (GP) surgeries/clinics and public spaces, mailing lists and via online/social media.

Consent will be taken separately for the run-in phase and randomised trial. Written informed consent will be obtained before the participant undergoes any research procedures, including screening tests. The lead consultant at each centre will identify CYP who are eligible and provide their details to the recruiting research nurses, who will provide the information sheets to the parents/legal guardians and the participant. The family will be given sufficient time to decide whether they are interested and would like to take part. The nurse will contact the family to answer any questions and provide clarity.

The first participant was recruited to the run-in phase on 23 April 2021 and the first randomisation took place on 23 July 2021. Recruitment to the RCT phase will end 31 January 2025, as this phase lasts for 1 year, followed by a 6-month study close-out period, the expected trial completion date is 31 July 2026.

### Interventions

Omalizumab dose and administration will be determined by the CYP’s weight and serum IgE level (75–600 mg) delivered through 1–4 subcutaneous injections either two or four weekly for 48 weeks depending on dose required according to the manufacturer’s dosing schedule.^12^ For CYP with a serum IgE level lower than the current recommended range (IgE<30 IU/mL), the lowest dose of omalizumab (75 mg four weekly) will be prescribed, and for those with an IgE level higher than the recommended range (>1500 IU/mL), the highest dose recommended by the manufacturer (600 mg two weekly) will be prescribed.

Mepolizumab dose will be determined by age (40 mg 6–11 years or 100 mg≥12 years) and administered as a single injection 4 weekly for 48 weeks from prefilled syringes.

Administration of investigational medicinal products (IMPs) will be recorded in the medical records as per routine care and monitored as part of on-site study monitoring procedures.

CYP may discontinue study treatment if: requested by the participant or family; adverse event (including allergic reaction to IMP) that has resulted from treatment administration, where the investigator considers that it would not be safe for the patient to continue treatment; eligibility violation; investigator considers a participant’s health will be compromised; and the trial is terminated.

All CYP will continue their maintenance asthma therapy and will continue to use their prescribed reliever therapy for acute symptoms. The dose and regimen of maintenance therapy can be adjusted by the clinical team as deemed necessary at any time. If CYP develop worsening asthma control, they will seek help from healthcare services as usual and according to their asthma management plan.

### Primary outcome and estimand

The primary outcome is the number of asthma attacks over 52 weeks. An asthma attack is defined as requiring high dose systemic steroids (oral, intravenous, intramuscular) or hospital admission (≥4 hours in the hospital).

#### The primary estimand

We aim to estimate the treatment effect for CYP as allocated to their original treatment even if they have discontinued treatment, but we will not include the effect of taking an alternative potentially highly effective biologic. The summary measure will be adjusted between arm incident rate ratio along with the 95% credible intervals, and we will report the posterior probability for mepolizumab to be non-inferior to omalizumab. The estimand is specified using the five attributes recommended in ICH E9 addendum.^13^ Full specification of the estimand can be found in online supplemental table 1.

### Secondary outcomes

1. Composite Asthma Severity Index (CASI) at weeks 4, 16, 32 and 52.^14^
2. Paediatric Asthma Quality of Life Questionnaire (mini-PAQLQ) Score—at weeks 4, 16, 32 and 52.
3. Lung function (FEV_1_, bronchodilator reversibility) at weeks 4, 16, 32 and 52.
4. Fractional exhaled nitric oxide (FeNO)—four weekly.
5. ACT or cACT Score—four weekly.
6. Inhaled corticosteroid daily dose—four weekly.
7. Sputum inflammatory cell count and eosinophil peroxidase—at weeks 4, 16, 52.
8. Health outcome measured by EuroQuol 5 Dimension (EQ-5D) Visual Analogue Scale (EQ-5D-Y) at weeks 4, 16, 32 and 52.
9. Adverse events.
10. Adverse events of special interest (anaphylaxis, hypersensitivity reaction, respiratory infections, upper and lower respiratory infection, injection site reactions, headache, nausea, and aches and pains).

### Participant timelines

CYP will attend four weekly visits (or two weekly if determined by omalizumab dosing) for at least 16 weeks, and thereafter at weeks 32 and 52. Subsequently, for families who agree to be trained, injections will be administered at home during directly observed therapy on video calls as home care, until treatment is completed at week 48. Children aged 6–11 years, and randomised to receive mepolizumab, will continue to attend in-person as the 40 mg dose is not available for home administration. Assessment at the four weekly treatment visits will include: adverse event’s assessment, asthma attack history, current medications, ACT/cACT, administration of omalizumab or mepolizumab and medication changes.

In-person visits at weeks 4, 16, 32, 52 will include vital signs including oxygen saturation, respiratory rate, wheeze on auscultation, height, weight and body mass index. Additional assessments at these time points will include CASI, mini-PAQLQ, EQ-5D, full blood count including eosinophils, induced sputum for inflammation (optional), FeNO, adherence monitoring data, eosinophil peroxidase, urine (metabolomics), nasal and oropharyngeal swabs (microbiome), breath samples (optional). See online supplemental figure 2 for full visit schedule.

### Randomisation and blinding

CYP will be allocated to treatment arm using minimisation with a random component that provides a 90% chance to choose the group that would allow more balance between the groups as recommended.^15^ Minimisation will be performed using algorithm programmed in an online system where the stratification variables are: centre, blood eosinophils (<300/≥300 per µL),^16^ IgE (<30, 30–1500, >1500 IU/mL) and type of asthma (RDA/STRA). This trial is open-label and the study participants and study team are not blind to treatment intervention, but all study team members, apart from the trial statistician, statistical supervisor and the data monitoring committee will not see any accrued data grouped by arm.

### Data collection

Trial data will be collected on an electronic case report form. The principal means of data collection from patient visits will be electronic data capture (EDC) via the internet using the OpenClinica database. Outcome data will be collected for all CYP regardless of treatment adherence unless they actively withdraw from data collection. All data will be recorded in the EDC by clinical and research staff and will be signed off by centre designee. All changes made following initial submission of data will have an electronic audit trail with a date.

### Statistical methods

#### Sample size

The trial concerns an important but rare subgroup of severe asthma patients, and recruitment is anticipated to be highly challenging. Consequently, the trial has been designed around a maximum feasible sample size, meaning a sample size we can recruit using an extended network of centres over a timely period. The trial uses a Bayesian framework to incorporate existing information, and we have undertaken simulations to explore what could be demonstrated with this fixed and limited sample size. This is presented in terms of the probability of NI for three scenarios, that is, if mepolizumab is inferior, the same as, or superior to omalizumab. These results were presented to the trial funder (National Institute for Health Research, Efficacy and Mechanism Evaluation Panel who agreed that there was value in undertaking a trial of this size.

CYP with PSA are expected to have a minimum of four asthma attacks in the previous year, or at least one PICU admission.^17^ A NI margin of 0.5 attacks per year was selected after discussion with clinicians and parents. A reduction of one attack per year was reasoned to be the minimum benefit required given 12 months of injections with either treatment, and the primary NI margin was taken to be half the minimum benefit required. We demonstrate the value of the trial based on a primary NI margin.

To calculate the maximum feasible sample size, we undertook a survey of 11 specialist paediatric severe asthma centres in the UK originally identified to take part in this trial. These centres combined have 170 new annual PSA referrals, and each centre had an existing cohort of approximately 50 eligible children. Over a 3-year period, we estimated there will be 1060 children with PSA to be eligible for invitation to the run-in study. Assuming a 50% acceptance rate, based on previous experience in this population and parent representative group feedback, we estimate n≈500 will be recruited to the run-in study. Pilot data show approximately 30% of PSA will have STRA, and 15% have RDA giving 225 eligible CYP.^18^ Assuming a recruitment rate of 66% of these CYP (reasoned on their commitment to the run-in study and the severity of their condition), we anticipate the feasible maximum will be 150 children in the randomised trial. The estimated withdrawal rate is unlikely to be higher than 15% (seen in a 48-week trial where children had to cross-over treatments).^19^ We therefore estimate that we will obtain 130 CYP who have full (52 weeks) follow-up data.

12 scenarios were explored to examine the strength of evidence this trial may provide under three potential outcomes (mepolizumab is better, no different or worse than omalizumab) using a sample size of 130 (n=65 per arm). Results based on 1000 simulations were repeated to indicate what would be expected for 75% of the sample size and introducing overdispersion in the outcome. The summarised simulation results by calculating the average posterior probability of being non-inferior for a 0.5 NI margin demonstrate that a trial of this size is likely to provide results that would be convincing to change prescribing practice. More information on the simulation results can be seen in online supplemental tables 3 and 4.

#### Analysis

The trial results will be reported according to Consolidated Standards of Reporting Trials (CONSORT) and the CONSORT extension for NI and equivalence randomised trials.^20 21^

We will use a Bayesian analysis for the primary outcome only and this will include informative clinician elicited priors on the log treatment effect and log attack rate on the omalizumab arm. More information on the clinician elicitation and the results of this activity can be found in online supplemental material section 8. We will also examine the use of alternative priors on the mean exacerbation rate and the treatment effect. Emerging randomised evidence during the trial will be used to provide an update to the clinician elicited prior as a sensitivity analysis. The first supplementary analysis on the primary outcome (treatment policy approach) will also be undertaken in the Bayesian framework. All other analyses on primary and secondary outcomes will be undertaken in the frequentist framework. In analysis of secondary outcomes, focus will be on estimation of the treatment effect rather than testing NI hypothesis as the NI margins have not been prespecified.

The primary analysis of 52-week asthma attack rate will be done on the modified ‘while on treatment’ population, which will include all randomised CYP who receive at least one dose and up to the time they complete the study, withdraw consent from the study or take another biologic to that which they were allocated (either through switching between arms or new biologic). As a consequence, there will be no multiple imputation for the primary outcome analysis. The analysis population sets for supplementary analysis can be seen in online supplemental table 5.

A Bayesian Poisson regression model will be used to model the primary outcome with treatment arm and minimisation stratification variables (centre, blood eosinophils (<300/≥300 per µL) and IgE (<30, 30–1500, >1500 IU/mL), type (refractory DA/STRA) included as covariates. The recruitment site will be included as a random effect unless there are fewer sites than expected or another reason to model the site as a fixed effect.^22^

Follow-up time will be included as an offset term to model the rate of exacerbations. Follow-up time will be calculated from the time of randomisation to the CYP’s last study visit (regardless of treatment discontinuation for any reason but in absence of other biologics). A negative binomial regression will replace the Poisson model if there is evidence for over dispersion, which will be checked by fitting a negative binomial and examining the overdispersion parameter. We will report the probability of mepolizumab being non-inferior to omalizumab, as well as the IRR and 95% credible intervals.

Based on the results of the elicitation workshop, a Gamma distribution with parameters (6.36, 4.5) was selected as a final prior for parameter 1; the mean of exacerbation rate in omalizumab arm, exp(α); and a normal distribution (mean=−5.05, SD=12) was confirmed as a prior distribution for parameter 2; the per cent change in exacerbation rate between mepolizumab and omalizumab, expressed as (exp(β1)−1)×100 or incidence rate ratio (IRR−1)×100 (see online supplemental figures 3 and 4). rjags V.4-12 will be used to include the priors in the primary analysis model.

Three supplementary analyses on the primary outcome will be performed: (1) to estimate the treatment effect targeting the treatment policy estimand, (2) to estimate the treatment effect in CYP that adhered to the treatment they were assigned and (3) to estimate the treatment effect if other biologics were not available and CYP did not cross over to the other treatment arm.

All secondary efficacy outcomes are continuous and have been measured repeatedly. As a result, we will fit a mixed effect linear regression model with random subject and centre effects, minimisation stratification variables and time. We will use the same analysis population as the primary analysis.

The baseline randomisation season of CYP will also be included a categorical variable with four levels defined as September–November; December–February; March–May and June–August. NI will not be the focus of these analyses, but we aim to estimate the adjusted mean difference between treatment arms with a 95% CI. A time-by-arm interaction will be included to obtain estimates for mean differences at 4, 16, 32 and 52 weeks. Models will be fitted using restricted maximum likelihood and assumptions will be examined using residual analysis, including the examination of graphical displays such as normal quantile plots as recommended to provide unbiased and robust variance parameter estimates. For CASI, FEV1 and QoL outcomes, we will plot model results over time with 95% CIs by the arm.

We will undertake subgroup analysis based on the type of asthma (STRA and refractory DA) and ethnicity by including an interaction between these variables and treatment arm into the model. Since we are not powered to conduct hypothesis testing in subgroups, the findings will be presented using forest plots and will serve as a basis for generating hypotheses rather than drawing definitive conclusions.

Adverse events will be coded using Medical Dictionary for Regulatory Activities and will be summarised at the Preferred Term and System Organ Class levels. AEs will be tabulated by arm and severity for the number of CYP with at least one adverse event, presented as frequencies and proportions, and number of events occurring among all CYP, presented as counts, mean (SD) number of events per participant and incident rates. Calculation of proportions will use denominators per the safety population definition, and incident rates will use total CYP follow-up time to account for differential follow-up.

For counts that are reasonably large at the system organ class level, we will estimate the IRR and 95% CIs using a negative Binomial model or suitable model, adjusting where possible for minimisation stratification variables. The results from these models will be presented graphically along with the raw counts using visual approaches such as dot plots.^23^

Mechanistic analysis will be undertaken to explore whether baseline serum IgE and baseline blood/BAL/sputum eosinophils are associated with treatment benefit (measured using both CASI and then asthma exacerbations count) using within arm and between arm modelling and suitable generalised linear models.

### Trial oversight

The trial will be overseen by a trial steering committee (TSC), which will comprise of at least one independent chair, statistician and clinician and one study team member for each two independent members. The TSC will supervise the conduct and progress of the trial. The Trial Management Group (TMG) will be composed of the chief investigator and key collaborators, community representative, trial statistician and trial manager. The TMG will be responsible for the day-to-day conduct of the trial. A fully independent data monitoring and ethics committee (DMEC) will be set up to monitor progress, child safety and any ethical issues involved in this trial through regular 6-monthly interims without any formal statistical rules. The DMEC will comprise of at least two clinicians and one statistician.

### Confidentiality

Confidentiality will be maintained by use of subject ID number for all study documents and central data and staff will comply with the requirements of the Data Protection Act 2018 and the EU General Data Protection Regulation and will uphold the Act’s core principles.

### Patient and public involvement (PPI)

Asthma and Lung UK were included as a coapplicant on TREAT and as part of their role they recruit and facilitate a patient advisory group consisting of 2–4 parents or parent/child pairs. The group meets approximately every 3–6 months to discuss trial design, advise on recruitment strategies, retention, engagement and advertising. Recommendations so far have included valuable input into trial design (parents/carers and children did not favour a placebo arm, and preferred an open-label trial), primary research question, visit schedule, website design, advertising strategies and clarity of informed consent forms and patient information sheets.

## Ethics and dissemination

The study has been approved by the South Central—Berkshire Research Ethics Committee (REC) REC Number 19/SC/0634 and had Clinical Trials Authorisation from the Medicines and Helathcare Products Regulatory Agency (MHRA) (EudraCT 2019-004085-17). All parents/legal guardians will give informed consent for their child to participate in the trial (see online supplemental materials, eg, consent form), and CYP will give assent to participate. REC approval was received prior to shipment of IMP and enrolment of subjects. There have since been seven substantial amendments approved by the REC, making updates to the protocol including additional sites, the use of advertising and direct recruitment via the website, and other changes to adherence monitoring and/or minor protocol changes. Annual progress reports are submitted to the REC which include details of all serious adverse events (SAEs) recorded.

Results will be published in a peer-reviewed journal and presented at national and international conferences. Our PPI collaborators will support public engagement and dissemination activities to a wider PPI audience. This includes via the Asthma & Lung UK social media channels, newsletters, via the Imperial College Biomedical Research Centre, and each site’s PPI groups. De-identified data from the trial for the primary and secondary analysis will be available on request from the chief investigator after the trial has closed and all exploratory analysis has been completed.

## Discussion

This trial will provide much needed evidence for the efficacy of mepolizumab compared with omalizumab in CYP with true STRA and refractory DA. To our knowledge, this is the first trial directly comparing the efficacy of one biologic to another in severe asthma in either adults or CYP. Moreover, we are unaware of other trials of biologics that have undertaken a prolonged period of objective adherence monitoring to maintenance inhaled therapy during run-in to distinguish patients with STRA from those who we know may not be taking their maintenance treatment but require a biologic because of risk of severe asthma attacks (CYP with refractory DA).^24^ A further novelty includes randomisation to a biologic without considering the recommended prescribing criteria for biomarkers (either serum IgE for omalizumab or blood eosinophils for mepolizumab). This approach will enable an assessment of the optimal biomarkers that might predict a clinical response for each biologic in CYP. Additional mechanistic components include the assessment of eosinophil activation by quantifying eosinophil peroxidase, rather than numbers of blood eosinophils alone, to determine likely response to therapy.

The limitations include an open-label design, but this could not be avoided given the very different dosing regimens for each biologic. Another challenge has been relatively slow recruitment because of the COVID-19 pandemic, which resulted in a significant reduction in the number of asthma attacks children had because of social isolation, reduced mixing, and thus fewer viral infections, which are the main cause of exacerbations in this age group. It was decided that the minimum number of four attacks in the previous year was needed to assess efficacy, as this remains the current cut-off before biologics can be prescribed, but this reduced the number of eligible children significantly for at least 3 years.

The results of this trial will allow more rational, evidence-based prescribing of either omalizumab or mepolizumab to improve outcomes for CYP with PSA.

## supplementary material

10.1136/bmjopen-2024-090749 online supplemental file 1

10.1136/bmjopen-2024-090749 online supplemental file 2

## References

[1] Bloom CI, Saglani S, Feary J (2019). Changing prevalence of current asthma and inhaled corticosteroid treatment inthe UK: population-based cohort 2006-2016. Eur Respir J.

[2] Walsh LJ, Wong CA, Cooper S (1999). Morbidity from asthma in relation to regular treatment: a community based study. Thorax.

[3] Mukherjee M, Stoddart A, Gupta RP (2016). The epidemiology, healthcare and societal burden and costs of asthma in the UK and its member nations: analyses of standalone and linked national databases. BMC Med.

[4] Smith DH, Malone DC, Lawson KA (1997). A national estimate of the economic costs of asthma. Am J Respir Crit Care Med.

[5] Levy ML (2015). The national review of asthma deaths: what did we learn and what needs to change?. Breathe (Sheff).

[6] National Institute for Health and Care Excellence (NICE) (2017). Asthma: diagnosis and monitoring of asthma in adults, children and young people.

[7] Romano C (2015). Omalizumab therapy for children and adolescents with severe allergic asthma. Expert Rev Clin Immunol.

[8] Cabon Y, Molinari N, Marin G (2017). Comparison of anti-interleukin-5 therapies in patients with severe asthma: global and indirect meta-analyses of randomized placebo-controlled trials. Clin Exp Allergy.

[9] Jackson DJ, Bacharier LB, Gergen PJ (2022). Mepolizumab for urban children with exacerbation-prone eosinophilic asthma in the USA (MUPPITS-2): a randomised, double-blind, placebo-controlled, parallel-group trial. Lancet.

[10] Pavord ID, Korn S, Howarth P (2012). Mepolizumab for severe eosinophilic asthma (DREAM): a multicentre, double-blind, placebo-controlled trial. Lancet.

[11] Chung KF, Wenzel SE, Brozek JL (2014). International ERS/ATS guidelines on definition, evaluation and treatment of severe asthma. Eur Respir J.

[12] National Institute for Health and Care Excellence, BNF Omalizumab. https://bnf.nice.org.uk/drugs/omalizumab/.

[13] International Council for Harmonisation of Technical Requirements for Pharmaceuticals for Human Use Addendum on estimands and sensitivity analysis in clinical trials to the guideline on statistical principles for clinical trials. https://database.ich.org/sites/default/files/E9-R1_Step4_Guideline_2019_1203.pdf.

[14] Wildfire JJ, Gergen PJ, Sorkness CA (2012). Development and validation of the Composite Asthma Severity Index--an outcome measure for use in children and adolescents. J Allergy Clin Immunol.

[15] (1999). ICH Harmonised Tripartite Guideline. Statistical principles for clinical trials. International Conference on Harmonisation E9 Expert Working Group. Stat Med.

[16] Ortega HG, Yancey SW, Mayer B (2016). Severe eosinophilic asthma treated with mepolizumab stratified by baseline eosinophil thresholds: a secondary analysis of the DREAM and MENSA studies. Lancet Respir Med.

[17] Chipps BE, Haselkorn T, Paknis B (2018). More than a decade follow-up in patients with severe or difficult-to-treat asthma: The Epidemiology and Natural History of Asthma: Outcomes and Treatment Regimens (TENOR) II. J Allergy Clin Immunol.

[18] Sharples J, Gupta A, Fleming L (2012). Long-term effectiveness of a staged assessment for paediatric problematic severe asthma. Eur Respir J.

[19] Lemanske RF, Mauger DT, Sorkness CA (2010). Step-up therapy for children with uncontrolled asthma receiving inhaled corticosteroids. N Engl J Med.

[20] Schulz KF, Altman DG, Moher D (2010). CONSORT 2010 statement: updated guidelines for reporting parallel group randomised trials. BMJ.

[21] Piaggio G, Elbourne DR, Altman DG (2006). Reporting of noninferiority and equivalence randomized trials: an extension of the CONSORT statement. JAMA.

[22] Pedroza C, Truong VTT (2017). Estimating relative risks in multicenter studies with a small number of centers - which methods to use? A simulation study. Trials.

[23] Phillips R, Cro S, Wheeler G (2022). Visualising harms in publications of randomised controlled trials: consensus and recommendations. BMJ.

[24] Cook J, Beresford F, Fainardi V (2017). Managing the pediatric patient with refractory asthma: a multidisciplinary approach. J Asthma Allergy.

