# Supplementary material for: Treating severe paediatric asthma with mepolizumab or omalizumab: a protocol for the TREAT randomised non-inferiority trial
Source: BMJ Open. 2024 Aug 21;14(8):e090749. doi: 10.1136/bmjopen-2024-090749 (PMC11340717; doi:10.1136/bmjopen-2024-090749)
Supplement: online supplemental file 1 [file bmjopen-14-8-s001.pdf]

**TREAT Supplementary Materials**

# Table of Contents

1. Complete Inclusion and Exclusion Criteria ... 2
   PARTICIPANT ENTRY INTO RUN IN STUDY ... 2
      Inclusion criteria for run-in phase ... 2
      Inclusion criteria for RCT phase ... 3
      Exclusion criteria for run-in and RCT phase ... 3
2. Primary Estimand ... 4
3. Study Flow Chart ... 5
4. Primary and supplementary estimands ... 6
5. Visit Schedule ... 7
6. Sample size ... 9
7. Analysis populations ... 12
8. Summary of approach to obtain clinician elicited prior ... 13

# 1. Complete Inclusion and Exclusion Criteria

## PARTICIPANT ENTRY INTO RUN IN STUDY

Study Population: Children with severe asthma will be recruited from specialist paediatric severe asthma centres in the UK.

## Inclusion criteria for run-in phase

1. Written informed consent
2. Children aged 6 – 16 years
3. Confirmed diagnosis of asthma*
4. Poor asthma control** despite being prescribed high dose therapy***[8]

***Confirmation of asthma diagnosis, documented wheeze plus one or more of:**

- Airway hyper-responsiveness confirmed by direct or indirect challenge
- Documented bronchodilator reversibility (≥12%)
- Recorded evidence of spontaneous variation in FEV1 (≥12%) or peak flow (≥20%) in the past year

****Definition of poor control, at least 1 of the following:**

- Recurrent severe asthma attacks in the past year (≥4 per year if on high dose inhaled corticosteroids OR ≥2 per year if on maintenance oral corticosteroids) requiring either asthma related hospital admission (>/=4 hours in hospital) or high dose systemic steroids
- A single PICU admission in the past year

*****Definition of high dose therapy, either of:**

- Maintenance inhaled corticosteroids (budesonide ≥800μg/day or fluticasone ≥500μg/day) or equivalent (as defined in the BTS/SIGN guidelines 2019) plus a long acting β2 agonist plus montelukast (or previous failed trial) or trial of other add on therapy such as theophylline
- Maintenance daily or alternate day oral corticosteroids

**Adherence to inhaled corticosteroids to be assessed during run-in and it will continue throughout the period of the intervention, for the full 52 weeks:**

All participants will undertake a period of adherence monitoring using electronic monitoring devices following enrolment in the study.

- Those with ongoing poor control (ACT / cACT<20) and monitored adherence of ≥80%* will be eligible for intervention study.
- Those with >/=1 attack (defined as need for high dose systemic steroids or hospital admission (>/=4 hours in hospital)) and monitored adherence of ≥80%* at the end of this period will be eligible for the intervention study.
- Those with ongoing poor control and monitored adherence of <80%* will enter a period of enhanced monitoring.

*** Monitored adherence of 70-80% after the initial monitoring period can be accepted as eligible for intervention study without a period of enhanced monitoring in the following circumstances:**

1. If accompanied by GP prescription uptake data of above 80%
2. If the smart inhaler device can be shown to be the reason for the lower % adherence and prescription uptake data is difficult to obtain. For example, if the daily dose is adequate but there are issues with the way adherence is recorded

as AM/PM, missing data or device malfunction. In these cases, the participant can enter the RCT phase without the additional prescription uptake data.

## Inclusion criteria for RCT phase

1. Written informed consent
2. Children aged 6-17 years
3. Confirmed diagnosis of asthma with:
   i) Persistent poor control*/ >/= 1 attack after adherence assessment with ≥80% adherence during run-in (STRA) (see section 5.2 for circumstances whereby 70-80% monitored adherence is also eligible for intervention study) **OR**
   ii) Persistent poor control* and poor adherence despite optimal efforts to improve adherence, including enhanced monitoring (Refractory DA)
4. Female patients capable of becoming pregnant** must agree to use hormonal contraception, intrauterine device, intrauterine hormone-releasing system, or to complete abstinence*** for the duration of the trial and up to 100 days after the last dose of IMP.

***Persistent poor control defined as at least one of the following:**

- Asthma Control Test (ACT) or Childhood Asthma Control Test (cACT) score of <20
- ≥1 severe attack requiring either asthma related hospital admission (>/=4 hours in hospital) or high dose systemic corticosteroids during the adherence monitoring period
- If entering RCT directly: Recurrent severe asthma attacks in the past year (≥4 per year if on high dose inhaled corticosteroids OR ≥2 per year if on maintenance oral corticosteroids) requiring either asthma related hospital admission (>/=4 hours in hospital) or high dose systemic steroids

*** Females capable of becoming pregnant are defined as: fertile, following menarche and until unless permanently sterile. Permanent sterilisation methods include hysterectomy, bilateral salpingectomy and bilateral oophorectomy.*

****Complete abstinence (defined as refraining from heterosexual intercourse) must be in line with the preferred and usual lifestyle of the participant. Barrier contraception, periodic abstinence (e.g. calendar, ovulation, symptothermal, postovulation methods), withdrawal and progestogen-only oral hormonal contraception where inhibition of ovulation is not the primary mode of action are not acceptable methods of contraception.*

## Exclusion criteria for run-in and RCT phase

1. As a result of medical interview, physical examination or screening investigation the physician responsible considers the child unfit for the study or has a risk of non-compliance with study procedures.
2. Known hypersensitivity to Omalizumab or Mepolizumab or to any of the excipients.
3. The child has a history of drug or other allergy, which, in the opinion of the responsible physician, contra-indicates their participation.
4. Participant is female who is pregnant, lactating or within 6 weeks post-partum or breast feeding.
5. The child has participated within 3 months in a study using a new molecular entity, another study investigating drugs or in a study with invasive procedures.
6. Significant alternative diagnoses that may mimic or complicate asthma, in particular dysfunctional breathing, panic attacks, and overt psychosocial problems (if these are

thought to be the main diagnosis; they can be present in addition to asthma major problem rather than in addition to severe asthma)
7. Significant other primary pulmonary disorders in particular cystic fibrosis, or interstitial lung disease
8. Diagnosis of chronic inflammatory diseases other than asthma (e.g. inflammatory bowel disease)
9. The child has taken asthma-related biologics within 6 months

## 2. Primary Estimand

*Supplemental Table 1: Primary Estimand*

| Estimand attribute | Description |
|---|---|
| Population | Patients with confirmed diagnosis of STRA/ RDA meeting TREAT eligibility criteria. |
| Treatment condition(s) | Treatment with Mepolizumab compared to Omalizumab up to 52 weeks despite any treatment discontinuation but in the absence of other biologics. |
| Variable (outcome) | Asthma exacerbation rate up to 52 weeks; defined as the number of asthma attacks requiring high dose systemic steroids (oral, intravenous, or intramuscular) or asthma related admission to hospital (>/=4 hours in the hospital). |
| Strategies used to handle Intercurrent events | Cross-over between treatment arms – while on treatment[1]<br>Use of other biologics (Dupilumab) – while on treatment[1]<br>Study treatment discontinuation – treatment policy[2]<br>Use of maintenance oral steroids - treatment policy[2] |
| Population-level summary measure | Incident rate ratio over 52 weeks adjusted for stratification variables (centre, blood eosinophils (<300/≥300 per mcl) and IgE (<30, 30-1500, >1500IU/ml), type (RDA/STRA) |

[1] A modified 'while on treatment strategy' will estimate the treatment effect in those taking the original allocated biologic treatment OR while they have discontinued, i.e. excluding the impact of alternative potentially highly effective biologic.

[2] A treatment policy strategy considers the occurrence of the associated event as irrelevant, and participant data are analysed regardless.

## 3. Study Flow Chart

*Supplemental Figure 1: Run-In study design with estimated recruitment numbers: Extended run-in with adherence monitoring to identify children with severe therapy resistant asthma (STRA) and Refractory Difficult Asthma (Refractory DA)*

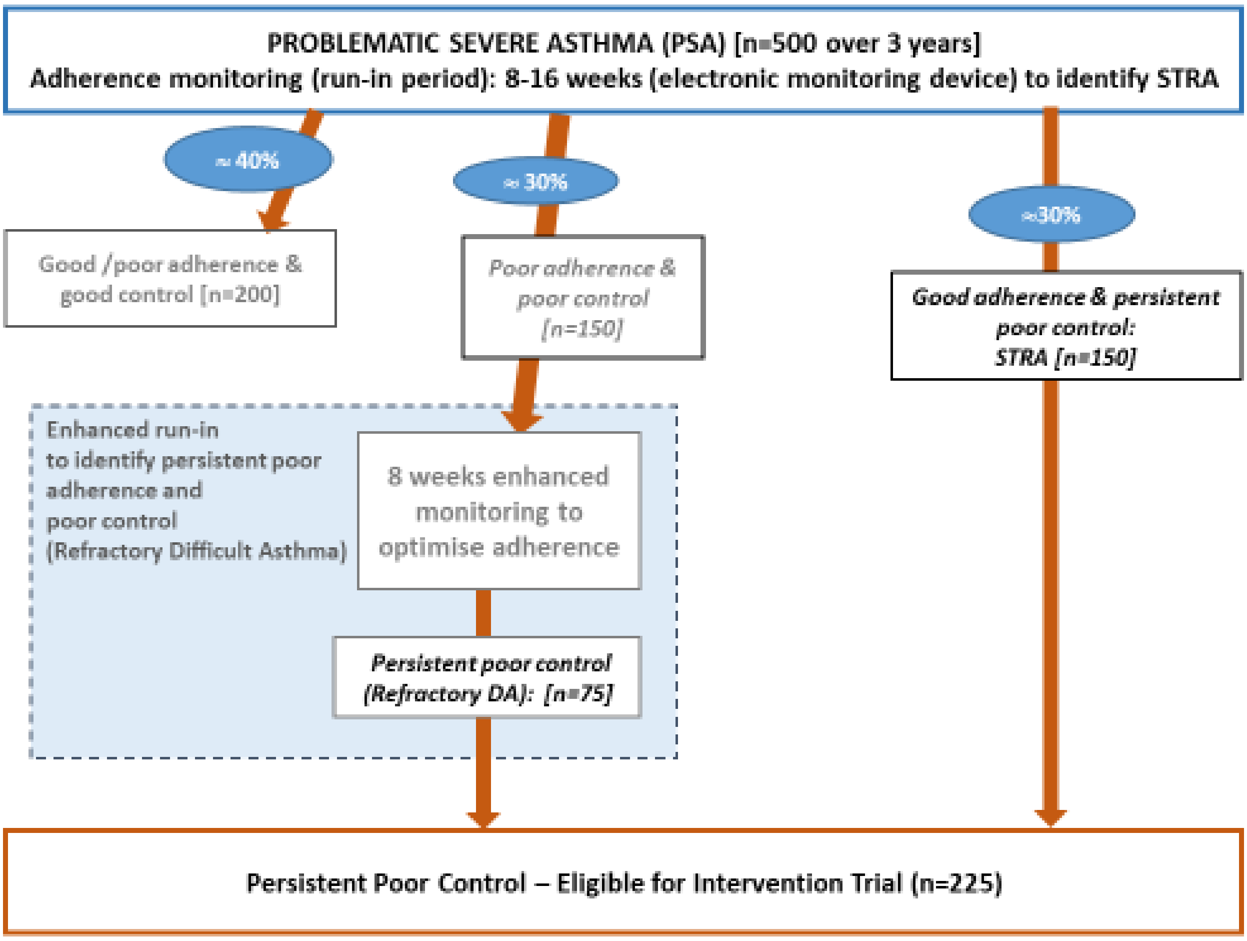

## 4. Primary and supplementary estimands

*Supplemental Table 2: Estimand for Primary Outcome*

| Attribute | Supplementary Estimand1: Treatment Policy | Supplementary Estimand2: Principal Stratum | Supplementary Estimand3: Hypothetical |
|---|---|---|---|
| Population | Patients with confirmed diagnosis of STRA/ RDA meeting TREAT eligibility criteria | Patients with confirmed diagnosis of STRA/ RDA meeting TREAT eligibility criteria | Patients with confirmed diagnosis of STRA/ RDA meeting TREAT eligibility criteria |
| Treatment condition(s) | treatment with Mepolizumab compared to Omalizumab up to 52 weeks | treatment with Mepolizumab compared to Omalizumab up to 52 weeks | treatment with Mepolizumab compared to Omalizumab up to 52 weeks |
| Variable (outcome) | Up to 52-week asthma exacerbation rate; defined as the number of asthma attacks requiring high dose systemic steroids (oral, intravenous, or intramuscular) or asthma related admission to hospital (>/=4 hours in the hospital). | Up to 52-week asthma exacerbation rate; defined as the number of asthma attacks requiring high dose systemic steroids (oral, intravenous, or intramuscular) or asthma related admission to hospital (>/=4 hours in the hospital). | Up to 52-week asthma exacerbation rate; defined as the number of asthma attacks requiring high dose systemic steroids (oral, intravenous, or intramuscular) or asthma related admission to hospital (>/=4 hours in the hospital). |
| Intercurrent events and the strategies used to handle them in the analysis | Cross-over between treatment arms – treatment policy | Cross-over between treatment arms – principal stratum (applies to both treatment arms) | Cross-over between treatment arms – hypothetical approach |
| | Use of other biologics (Dupilumab) – treatment policy | Use of other biologics (Dupilumab) – principal stratum (applies to both treatment arms) | Use of other biologics (Dupilumab) – hypothetical approach |
| | Study treatment discontinuation – treatment policy | Study treatment discontinuation – principal stratum (applies to both treatment arms) | Study treatment discontinuation – treatment policy |
| | Use of maintenance oral steroids - treatment policy | Use of maintenance oral steroids - treatment policy | Use of maintenance oral steroids - treatment policy |
| Population-level summary measure | Incident rate ratio over 52 weeks adjusted for stratification variables (centre, blood eosinophils (<300/≥300 per mcl) and IgE (<30, 30-1500, >1500IU/ml), type (RDA/STRA) | Incident rate ratio over 52 weeks adjusted for stratification variables (centre, blood eosinophils (<300/≥300 per mcl) and IgE (<30, 30-1500, >1500IU/ml), type (RDA/STRA) | Incident rate ratio over 52 weeks adjusted for stratification variables (centre, blood eosinophils (<300/≥300 per mcl) and IgE (<30, 30-1500, >1500IU/ml), type (RDA/STRA) |

# 5. Visit Schedule

*Supplemental Figure 2: Participant Visit Schedule*

| Visit | Screening[10,11] | Baseline adherence monitoring[1] (N = 500) | Follow up adherence monitoring visit[2] (N= 500) | Follow-up for enhanced adherence monitoring | Bronchoscopy visit[3,10] (N=150) | Randomised controlled trial (N=150; 75 vs 75) | | | | | | | | | | | | | |
|---|---|---|---|---|---|---|---|---|---|---|---|---|---|---|---|---|---|---|---|
| **Visit number** | 1 | 2 | 3 | 4 | 5 | 6 | 7 | 8 | 9 | 10 | 11 | 12 | 13 | 14 | 15 | 16 | 17 | 18 | 19 |
| **Time** | Day 1 | 0 – 28 days post screening | 8-16 wks post Visit 2 | 8-16 wks post visit 3 | Within 1-3 months of visit 3 or 4 | Wk 0 / Baseline[4,11] | Wk 4 | Wk 8 | Wk 12 | Wk 16 | Wk 20 | Wk 24 | Wk 28 | Wk 32 | Wk 36 | Wk 40 | Wk 44 | Wk 48 | Wk 52/ End of study |
| **Window** | | min 0d/max 28d (post visit 1) | min 56d/max 84d (post visit 2) | min 56d/max 84d (post visit 3) | min 0d/max 90d (post visit 3/4) | min 0d/max 14d (post visit 3/4/5) | 4 wks +/- 7d | 4 wks +/- 7d | 4 wks +/- 7d | 4 wks +/- 7d | 4 wks +/- 7d | 4 wks +/- 7d | 4 wks +/- 7d | 4 wks +/- 7d | 4 wks +/- 7d | 4 wks +/- 7d | 4 wks +/- 7d | 4 wks +/- 7d | 4 wks +/- 7d |
| Informed consent | X | | | | | X | | | | | | | | | | | | | |
| Algorithm for inclusion/exclusion | X | | X* | X | | X | | | | | | | | | | | | | |
| Pregnancy test (female post-pubertal pts)[5] | | | | | | X | | | | | | | | | | | | | |
| Randomisation | | | | | | X | | | | | | | | | | | | | |
| Adverse events | | X | X | X | X | X | X | X | X | X | X | X | X | X | X | X | X | X | X |
| Demographics | X | | | | | | | | | | | | | | | | | | |
| Asthma / exacerbation history | X | X | X | X | X | X | X | X | X | X | X | X | X | X | X | X | X | X | X |
| Past medical and drug history | X | | | | | | | | | | | | | | | | | | |
| Current medications | X | X | X | X | X | X | X | X | X | X | X | X | X | X | X | X | X | X | X |
| Physical examination | X | | | | | | | | | | | | | | | | | | |
| Vital signs (including oxygen saturation, respiratory rate, wheeze, BMI) | X | X | X | X | X | X | X | X | X | X | X | X | X | X | X | X | X | X | X |
| cACT/ACT[6] | | X | X | X | X | X | X | X | X | X | X | X | X | X | X | X | X | X | X |
| Mini PAQLQ | | X | X | X | | X | X | | | X | | | | | | | | | X |
| CASI | | X | X | X | | X | X | | | X | | | | X | | | | | X |
| Visual analogue score | | | | | | | X | | | X | | | | X | | | | | X |
| IMP (omalizumab / mepolizumab)[7] | | | | | | X | X | X | X | X | X | X | X | X | X | X | X | X | |
| ***Pulmonary function testing*** | | | | | | | | | | | | | | | | | | | |
| Spirometry[13] | | X | X | X | | X | X | | | X | | | | X | | | | | X |
| BDR | | X | X | X | | X | X | | | X | | | | X | | | | | X |
| ***Labs*** | | | | | | | | | | | | | | | | | | | |
| Urine sample | | X | | | X | X | X | | | X | | | | | | | | | X |
| Saliva/urine for cotinine | | X | | | | X# | | | | | | | | | | | | | |
| Full Blood Count (inc. eosinophils) | | X | | | X | X | X | | | X | | | | | | | | | X |
| Vitamin D3 level | | X** | | | X | X*** | | | | | | | | | | | | | |
| Total IgE | | X | | | | X# | | | | | | | | | | | | | |
| Specific IgE RASTs[8 14] | | X** | | | | X#** | | | | | | | | | | | | | |
| Genotype (blood) | | X | | | | X# | | | | | | | | | | | | | |
| Other study bloods (additional serum, immunoCAP, transcriptomics) | | X | | | | X# | X | | | | | | | | | | | | |
| ***Inflammometry*** | | | | | | | | | | | | | | | | | | | |
| Sputum induction and processing | | | | | X | X*** | X | | | X | | | | | | | | | X |
| Exhaled nitric oxide (50ml/sec)[12] | | X | X | X | X | X | X | X | X | X | X | X | X | X | X | X | X | X | X |
| ***Other procedures*** | | | | | | | | | | | | | | | | | | | |
| Electronic monitoring device given | | X | | | | | | | | | | | | | | | | | |
| Adherence monitoring data | | | X | X | | | X | | | X | | | | X | | | | | X |
| Skin prick tests[9 14] | | X** | | | | X#** | | | | | | | | | | | | | |
| Bronchoscopy | | | | | X | | | | | | | | | | | | | | |
| Broncho-alveolar lavage | | | | | X | | | | | | | | | | | | | | |
| Endobronchial biopsy | | | | | X | | | | | | | | | | | | | | |
| Endobronchial brushings | | | | | X | | | | | | | | | | | | | | |
| Nasal brushings | | | | | X | | | | | | | | | | | | | | |
| Oropharyngeal swab | | | | | X | X**** | X | | | X | | | | | | | | | X |
| Nasal swab | | | | | X | X**** | X | | | X | | | | | | | | | X |
| Breath samples (eNOSE) | | X | | | | X | X | | | X | | | | | | | | | X |

## Notes:

1. Screening and baseline visits can take place on the same day, in which case assessments do not need to be repeated.

2. Participants with poor control and poor adherence at the end of the monitoring study may undergo one further period of enhanced monitoring; baseline and follow-up assessments will be repeated.

3. Only those participants with ongoing poor control despite good adherence proceed to the RCT phase.

4. Visits at 12, 20, 24, 28, 36,40, 44 and 48 weeks can be done remotely. The first 3 injection visits (baseline, wk 4, wk8) and then weeks 16, 32 and 52 visits are mandatory to take place in hospital.

5. When menarche occurs after the randomisation visit, a pregnancy test will be performed before the drug is administered to the patient.

6. cACT will be continued throughout the study even if the child's age goes over 12 years

7. Some participants randomised to Omalizumab will receive 2 weekly injections. Study drug will be given, vital signs observed, AEs tracked.

8. RASTs: house dust myte, cat, dog, birch pollen, mixed grass, tree pollen, peanut, milk, egg, mixed mould.

9. Skin prick test: house dust myte, cat, dog, birch pollen, tree, mixed grass, mixed mould.

10. If a patient had smartinhaler monitoring as part of standard of care and bronchoscopy is clinically indicated (and was not done within the last 12 months), screening and bronchoscopy visits can take place on the same day in which case assessments do not need to be repeated.

11. If a patient had smartinhaler monitoring as part of standard of care and bronchoscopy is not clinically indicated, screening and wk 0 visits can take place on the same day in which case assessments do not need to be repeated.

12. Exhaled Nitric Oxide assessments not required for visits performed remotely

13. If Spirometry cannot be performed at a visit, handheld Spirometers can be used

14. Specific IgE RASTs and/or skin prick tests

* Assessment only performed if the child will not undergo a further period of enhanced monitoring.

** Tests can be used from the last 6 months if available.

*** Tests only repeated for children that didn't have Bronchoscopy/Visit 5.

**** If not taken during bronchoscopy visit.

# If child skipped Run-in phase

**Key for costing categories**

| | |
|---|---|
| Standard treatment | |
| Treatment / Excess treatment cost | |
| Support cost | |
| Research cost | |

## 6. Sample size

### Sample size rationale

The trial concerns an important but rare sub-group of severe asthmatic patients and recruitment is anticipated to be highly challenging. A frequentist approach to a non-inferiority trial of mepolizumab to omalizumab would result in an unfeasibly large sample size. Rather than not undertake the trial at all, the evidence will be evaluated in a Bayesian analytical framework. The planned sample size has been based on what is possible to recruit nationally in a feasible time scale and budget.

As a consequence, the trial has been designed around a maximum feasible sample size, meaning a sample size we can recruit using an extended network of centres over a timely period. The trial uses a Bayesian framework to incorporate existing information and we have undertaken simulations to explore what could be demonstrated with this fixed and limited sample size. This is presented in terms of the probability of non-inferiority for three scenarios, i.e. if mepolizumab is inferior, the same as or superior to omalizumab. These results were presented to NIHR EME funders who decided that there was value in undertaking a trial of this size. A non-inferiority margin of 0.5 asthma attacks was selected as described in the main methods section of the paper.

To calculate the maximum feasible sample size we undertook a survey of 11 specialist paediatric severe asthma centres in the UK centres originally identified to take part in this trial. These centres combined have 170 new annual PSA referrals and each centre had an existing cohort of ≈50 eligible children. Over a 3-year period we estimated there will be 1,060 children with PSA to be eligible for invitation to the run-in study. Assuming a 50% acceptance rate, based on previous experience in this population and PPI group feedback, we estimate n≈500 will be recruited to the run-in study. Pilot data show approximately 30% of PSA will have STRA, and 15% have RDA giving 225 eligible children. Assuming a recruitment rate of 66% of these patients (reasoned on their commitment to the run-in study and the severity of their condition), we anticipate the feasible maximum will be 150 children in the randomised trial. The estimated withdrawal rate is unlikely to be higher than 15% (seen in a 48-week trial where children had to cross-over treatments)[(1)]. We therefore estimate 130 children case is a full (52-week) follow-up for 130 children.

In **Error! Reference source not found.** twelve scenarios examining three potential outcomes ( mepolizumab is better, no different or worse than omalizumab) are presented to indicate the strength of evidence this study may provide. Results are based on 1000 simulations using a sample size of 130 (n=65 per arm). Simulations were repeated to indicate what would be expected for 75% of the sample size and introducing overdispersion in the outcome

Data were simulated using a Poisson distribution with vague Gaussian prior distributions for the log rate in change (mean 0, SD 10), and log baseline rate with baseline of 3 and 2.5 exacerbations (mean 1.1 and 0.92 respectively, SD 10). The scenarios included using a prior on β (the log treatment effect) strongly in favour of mepolizumab, strongly in favour of omalizumab and also a vague prior. The simulation results are summarised by calculating the average posterior probability of being non-inferior using a 0.5 NI margin.

The results in Supplemental Table 3 demonstrate that if mepolizumab was truly equal in efficacy or superior to omalizumab then a trial recruiting 150 participants (assuming n=130 with outcome data) has the potential for providing high posterior probabilities. The average ranged from 0.87 up to >0.99 depending on whether a vague or informative prior was used on the treatment effect coefficient. Probabilities this high provide reassurance that a trial of this size has the potential to inform treatment decisions if mepolizumab is not an inferior treatment. When the sample size is 75% of that targeted, then results in Supplemental Table 3 show the average posterior probability is reduced (ranging from 0.80 to 0.89) when mepolizumab was similar in efficacy to Omalizumab, suggesting with a smaller sample size, the trial would likely still be informative to current evidence. When mepolizumab was inferior, the average posterior probabilities were reassuringly low, all 0.14 for 100% sample size and 0.16 to 0.21 for 75% sample size. Note that these probabilities should not be compared to a typical type 1 error rate of 0.05 as standard in a frequentist setting as they are not the same. The results from the simulations where overdispersion was introduced into the outcome (Supplemental Table 4) demonstrate that we would expect to obtain lower probabilities with means of 0.71 to 0.83 if mepolizumab was equally effective to omalizumab. The lower range of these probabilities now arguably includes results that could be considered less convincing on which to change prescribing practice.

*Supplemental Table 3: Estimated average posterior probability based on 1000 simulations assuming a Poisson distribution*

| **Scenario (true treatment difference)** | **β Prior** | **α prior** | **Average probability of Non-Inferiority when n=130 (100% sample size)** | **Average probability of Non-Inferiority when n=98 (75% sample size)** | **Scenario** |
|---|---|---|---|---|---|
| Efficacy of new = established | vague | vague | 0.88 | 0.80 | 1 |
| | no difference | Established Rx mean rate | 0.89 | 0.83 | 2 |
| | sceptical | Established Rx mean rate | 0.87 | 0.83 | 3 |
| | optimistic | Established Rx mean rate | 0.91 | 0.89 | 4 |
| Efficacy of new > established | vague | vague | >0.99 | >0.99 | 5 |
| | no difference | Established Rx mean rate | >0.99 | >0.99 | 6 |
| | sceptical | Established Rx mean rate | >0.99 | >0.99 | 7 |
| | optimistic | Established Rx mean rate | >0.99 | >0.99 | 8 |
| Efficacy of new < established | vague | vague | 0.14 | 0.21 | 9 |
| | no difference | Established Rx mean rate | 0.14 | 0.20 | 10 |
| | sceptical | Established Rx mean rate | 0.14 | 0.16 | 11 |
| | optimistic | Established Rx mean rate | 0.14 | 0.22 | 12 |

*Supplemental Table 4: Estimated posterior probabilities based on 1000 simulations assuming a negative binomial distribution*

| **Scenario (true** | **β Prior** | **α prior** | **Average probability of Non-Inferiority** | **Average probability of Non-Inferiority when** | **Scenario** |
|---|---|---|---|---|---|

| treatment difference) | | | when n=130 (100% sample size) | n=98 (75% sample size) | |
|---|---|---|---|---|---|
| Efficacy of new = established | vague | vague | 0.72 | 0.69 | 1 |
| | no difference | Established Rx mean rate | 0.77 | 0.76 | 2 |
| | sceptical | Established Rx mean rate | 0.71 | 0.7 | 3 |
| | optimistic | Established Rx mean rate | 0.83 | 0.82 | 4 |
| Efficacy of new > established | vague | vague | 0.98 | 0.97 | 5 |
| | no difference | Established Rx mean rate | 0.99 | 0.98 | 6 |
| | sceptical | Established Rx mean rate | 0.98 | 0.97 | 7 |
| | optimistic | Established Rx mean rate | 0.99 | 0.99 | 8 |
| Efficacy of new < established | vague | vague | 0.33 | 0.32 | 9 |
| | no difference | Established Rx mean rate | 0.36 | 0.39 | 10 |
| | sceptical | Established Rx mean rate | 0.3 | 0.32 | 11 |
| | optimistic | Established Rx mean rate | 0.44 | 0.47 | 12 |

## 7. Analysis populations

*Supplemental Table 5: Trial analysis populations*

| |
|---|
| **PRIMARY population:** The ***modified 'while on treatment'*** population will include all randomised participants who receive at least one dose and up to the time they complete the study, withdraw consent from the study, or take another biologic to that which they were allocated (either through switching between arms or new biologic). |
| **Treatment Policy Population Set:** a modified intention-to-treat population including all participants who receive at least one dose of the assigned treatment regardless of then what treatments they take and discontinuation. |
| **Principal Stratum population Set:** This population will include all randomised participants who receive at least one dose of assigned treatment. It will taking account of study discontinuation through the analysis method Complier average causal effect (CACE). We will look at threshold ≥75%  for the assigned treatment under evaluation (i.e., 9 months). |
| **Hypothetical population Set:** The same starting population as the ***modified 'while on treatment'***. However, hypothetical strategy will be used to handle taking another biologic (either through switching between arms or new biologic). |

## 8. Summary of approach to obtain clinician elicited prior

We conducted a prior elicitation workshop using the Sheffield Elicitation Framework (SHELF)[(2)] and following the recommendations of Dallow et al[(3)]. This occurred after the trial had been funded. We used the roulette method for prior elicitation. The roulette method requires delegates to use "chips" to build a histogram to represent their beliefs about the true value of the unknown parameter. As it was not easy for clinicians to express their opinions based on the original parameters in our model ( $\alpha, \beta_1$), we asked their opinions about the transformation of parameters using the following questions:

a) The mean number of exacerbations for patients in the Omalizumab arm of the trial over 52 weeks

b) The relative treatment effect of Mepolizumab compared to Omalizumab during the study, expressed as a percentage

We constructed a detailed Prior Elicitation Plan (PEP) that documented the training of experts and the meeting processes. We also constructed an evidence dossier will all the relevant peer-reviewed research summarised.

Eight experts participated in the 2-day workshop in March 2021. Two weeks before the workshop, we shared the Training Document and the Evidence Dossier with the delegates and asked them to review the documents. On the first day of the workshop, we focused on reviewing concepts and warning of potential psychological biases we aim to avoid, and we also went through the training documents and test questions. Then we conducted a practical elicitation session with a hypothetical example.

The second day of the workshop started with reviewing the Evidence Dossier and discussing the relevant references, and then we ran Elicitation for two main parameters of the model. Two parameters were elicited, the mean 52-week exacerbation rate for children in the study population on omalizumab and the relative effect of mepolizumab compared to omalizumab expressed in percentages. The results of the elicitation can be seen in Supplemental Figure and Supplemental Figure . The mean of the Gamma distribution is approximately 1.4, indicating that the mean exacerbation rate over 52 weeks while on Omalizumab is 1.4. The mean of the percentage change is approximately -5%, suggesting a preference for Mepolizumab. This finding implies that, on average, Mepolizumab demonstrates a 5% reduction in mean exacerbation rates over 52 weeks compared to Omalizumab.

Priors will be updated with emerging randomised evidence during the trial conduct and the primary analysis will be based on the original and updated priors.

*Supplemental Figure 3: Final elicited prior for the mean 52-week exacerbation rate for children in the study population on omalizumab*

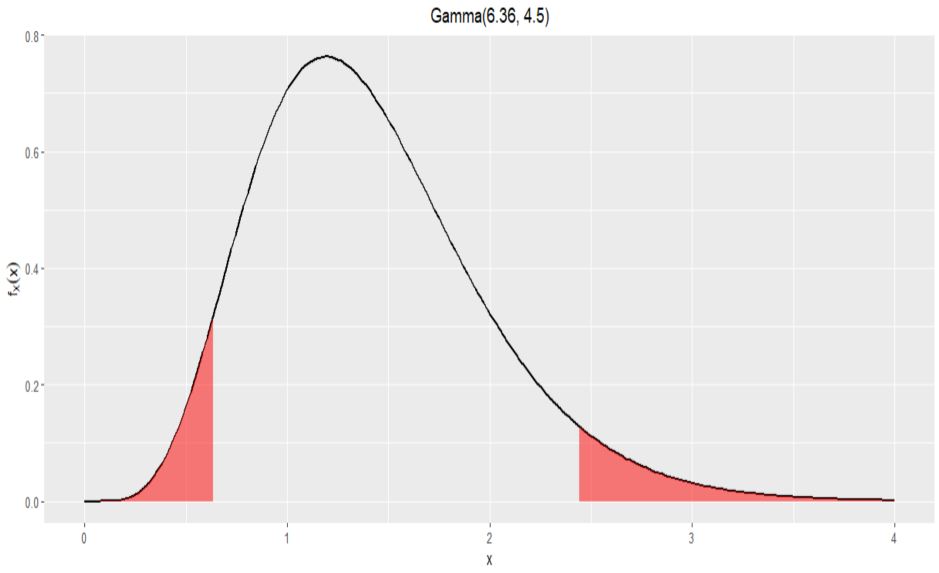


*Supplemental Figure 4: Final elicited prior for the relative effect of mepolizumab compared to omalizumab expressed in percentages*

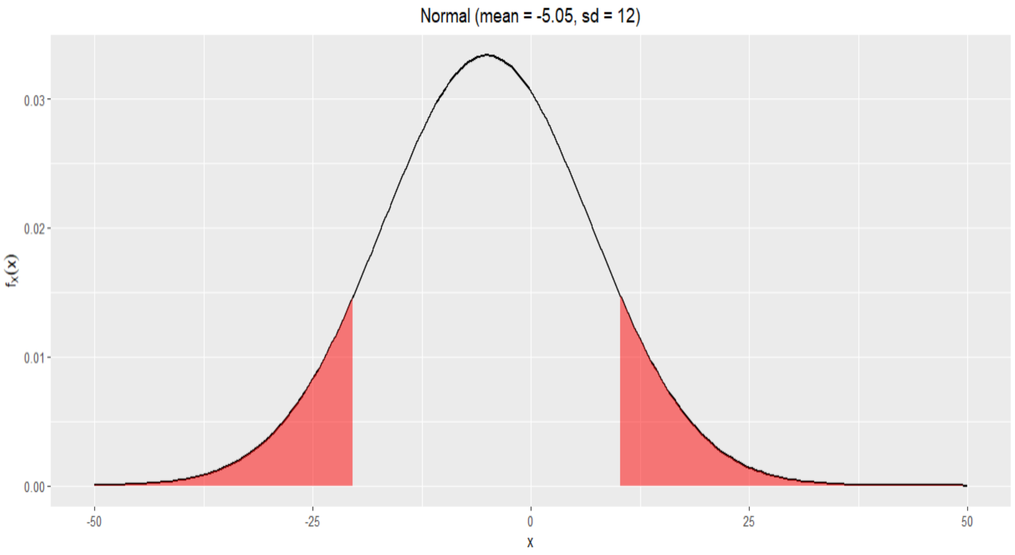

# References


1. Lemanske RFJ, Mauger DT, Sorkness CA et al. Step-up therapy for children with uncontrolled asthma receiving inhaled corticosteroids. *N Engl J Med* 2010: **362**:975–985.

2. Oakley J. E. and O'Hagan, A. SHELF: the Sheffield Elicitation  Framework (version 4). *School of Mathematics and Statistics, University of Sheffield, UK* Published Online First: 2019.http://tonyohagan.co.uk/shelf

3. Dallow N, Best N, Montague TH. Better decision making in drug development through adoption of formal prior elicitation. *Pharm Stat* 2018: **17**:301–316.
