# Supplementary material for: Treating severe paediatric asthma with mepolizumab or omalizumab: a protocol for the TREAT randomised non-inferiority trial
Source: BMJ Open. 2024 Aug 21;14(8):e090749. doi: 10.1136/bmjopen-2024-090749 (PMC11340717; doi:10.1136/bmjopen-2024-090749)
Supplement: online supplemental file 2 [file bmjopen-14-8-s002.pdf]

Imperial College London

[Insert Trust header here]

Chief Investigator: Professor Sejal Saglani

Study Protocol Number: 19IC5548

IRAS ID: 252084 **REC Ref:** 19/SC/0634

EudraCT: 2019-004085-17

Trial title: Treating severe paediatric asthma; a randomised controlled trial of mepolizumab and omalizumab; the TREAT trial

Parent/Carer Consent Form for RCT (Randomised Controlled Trial) phase

**Participant Trial ID Number:**	
**Child Name:**	
**Site Number/Name:**	
**Name of Principal Investigator:**	

Please <u>initial</u> each box if you agree with the following:

1 I confirm I have read and understood the Parent / Carer Information Sheet, Version ___ dated _ _ /_ _ /_ _ for the above study, have had enough time to review the information and have been able to ask questions which have been answered fully. ☐

2 I understand my child's participation is voluntary and I am free to withdraw consent at any time, without giving any reason, without my child's medical care or legal rights being affected. ☐

3 I understand my child's identity will never be disclosed outside of research and any information collected will remain confidential. ☐

1 copy for participant; 1 copy for Investigator Site File; 1 copy for participant notes

Imperial College London

TREAT

4	I understand that sections of any of my child's medical records and other personal data generated during the study may be examined by representatives of the Sponsor (Imperial College London), by people working on behalf of the Sponsor, or by representatives of regulatory authorities, or by representatives of other organisations involved in the study where it is relevant to taking part in this research. I give permission for these individuals to access my child's records.	☐
5	I consent to the use of my child's blood, urine and sputum, swabs, tissue samples for the analyses described in the Parent/Carer Information Sheet, some of which includes genetic analysis only for genes related to asthma.. I understand that if I withdraw my child from the study, my child's samples shall be destroyed if I request this, but the results collected from these samples can still be used.	☐
6	I give permission for my child's GP to be informed of participation in this study. ***(optional)***	☐
7	I agree for breath samples to be collected (eNOSE device) from my child. ***(optional)***	☐
8	I agree for results from previous bronchoscopy procedure my child has had (if performed during the last year) to be used for the study, and also for remaining samples collected during the procedure to be used for the study.	☐
9	I agree to share my contact details for the purpose of shipment of the study drug if a remote visit will be conducted.	☐
10	I agree to share an email address and my child's height, weight and ethnicity to calculate my child's lung function and also to transfer the test (spirometry) results to the research team if my child is given a hand-held lung function device.	☐
11	I agree to be contacted by the research team in future to get information about the outcome of my child's pregnancy should this occur.	☐

[Insert Trust header here]

Imperial College London

12 I agree to be asked for my education and employment status as needed for this trial. ☐

13 I agree to the storage and use of my child's samples for future ethically approved related research and I give permission for my child' samples to be sent to other organisations such as tissue bank and Imperial laboratory. ***(optional)*** ☐

14 I understand that the information collected about my child will be used to support other research in the future, and may be shared anonymously with other researchers.
(optional) ☐

15 I agree to be contacted in the future for research related to this study.
(optional) ☐

16 I agree for my child's data collected for the purpose of smartinhaler monitoring to be transferred and stored outside of UK and EU/EEA. The data transferred/stored will not allow the child to be identified. ☐

17 I agree for my child to participate in this study. ☐

______________________	______________________	______________________
**Name of Parent/Carer**	**Date**	**Signature**
**Name of Parent/Carer (optional)**	**Date**	**Signature**
**Name of Researcher taking consent**	**Date**	**Signature**
**Principal Investigator** *(if not Researcher taking consent)*	**Date**	**Signature**

1 copy for participant; 1 copy for Investigator Site File; 1 copy for participant notes
