# Supplementary material for: Treating severe paediatric asthma with mepolizumab or omalizumab: a protocol for the TREAT randomised non-inferiority trial
Source: BMJ Open. 2024 Aug 21;14(8):e090749. doi: 10.1136/bmjopen-2024-090749 (PMC11340717; doi:10.1136/bmjopen-2024-090749)
Supplement: Supplementary file 3 [file bmjopen-2024-090749.reviewer_comments.pdf]

PEER REVIEW HISTORY

BMJ Open publishes all reviews undertaken for accepted manuscripts. Reviewers are asked to complete a checklist review form (**http://bmjopen.bmj.com/site/about/resources/checklist.pdf**) and are provided with free text boxes to elaborate on their assessment. These free text comments are reproduced below.

ARTICLE DETAILS

**TITLE (PROVISIONAL)**	Treating severe paediatric asthma with mepolizumab or omalizumab: protocol for the TREAT randomised non-inferiority trial
**AUTHORS**	Cornelius, Victoria; Babalis, Daphne; Carroll, William; Cunningham, Steven; Fleming, Louise; Gaillard, Erol; Gupta, Atul; Janani, Leila; Kennington, Erika; Murray, Clare; Nagakumar, Prasad; Roberts, Graham; Seddon, Paul; Sinha, Ian; Streatfield, Claire; Weir, Elise; Saglani, Sejal

This article was not externally reviewed at BMJ Open. Protocols that have been independently assessed prior to submission to BMJ Open are usually fast-tracked to publication on the grounds that further substantial changes will not be possible. This independent assessment will usually be external, independent review for both a substantial grant award from a non-commercial or government funder and ethics approval.
